# Clinical Decision Support Systems in Indian Healthcare Settings: Benefits, Barriers, and Future Implications

**DOI:** 10.3390/healthcare13172220

**Published:** 2025-09-04

**Authors:** Shabeer Ali Thorakkattil, Sathvik Belagodu Sridhar, Suhaj Abdulsalim, Mohammed Salim Karattuthodi, Prashant Chandra, Mazhuvanchery Kesavan Unnikrishnan

**Affiliations:** 1 Pharmacy Services Department, Johns Hopkins Aramco Healthcare (JHAH), Dhahran 34465, Saudi Arabia; shabeer.thorakkattil@jhah.com; 2 Department of Pharmacy Practice and Clinical Pharmacy, Faculty of Pharmacy, Universiti Teknologi MARA (UiTM) Selangor Branch, Puncak Alam Campus, Puncak Alam 42300, Selangor, Malaysia; 3 RAK College of Pharmacy, RAK Medical and Health Sciences University, Ras Al Khaimah P.O. Box 11172, United Arab Emirates; sathvik@rakmhsu.ac.ae; 4 Department of Pharmacy Practice, College of Pharmacy, Qassim University, Buraydah 51452, Saudi Arabia; 5 Department of Pharmacy Practice, Manipal College of Pharmaceutical Sciences, Manipal Academy of Higher Education, Manipal 576104, Karnataka, India; salim.kt@manipal.edu; 6 Department of Pharmacy Practice, Amrita School of Pharmacy, Amrita Vishwa Vidyapeetham, Kochi 682041, Kerala, India; prashantc@pharmacy.aims.amrita.edu (P.C.); mkunnikrishnan@pharmacy.aims.amrita.edu (M.K.U.)

**Keywords:** clinical decision support systems, patient safety, health information technology, health informatics, electronic health records, public health

## Abstract

India’s vast and diverse population presents significant healthcare challenges owing to its scale, heterogeneity, and rapid growth. The Indian healthcare system, spanning the public, private, and non-profit sectors, shows marked inter-state variation in health indicators. Persistent gaps include variable quality of service, fragmented data, and uneven access to affordable care. Health information technology (HIT), particularly clinical decision support systems (CDSSs) integrated with electronic health records (EHRs), offers a path to more consistent evidence-based decisions. When implemented effectively, CDSSs can improve patient outcomes, reduce medical errors, and enhance quality through support for diagnosis, treatment, patient management, and prevention. Although India is rapidly adopting digital health tools, CDSS uptake remains limited because of infrastructure constraints, low awareness, data quality issues, integration challenges with EHRs, professional resistance, and insufficient training. Strategic action is required to overcome these barriers. Priorities include investment in robust IT infrastructure, comprehensive training programs, and public awareness initiatives, along with tighter integration of CDSSs with EHR platforms. With coordinated efforts by government agencies, healthcare institutions, and technology providers to address these barriers, India can leverage CDSSs to improve patient care and outcomes.

## 1. Introduction

India’s projected population of 1.5 billion by 2050, around 16% of the global total, poses significant healthcare challenges, particularly regarding access to quality care and the lag in adopting digital health technologies such as Clinical Decision Support Systems (CDSSs). Fragmented data systems, inadequate information technology (IT) infrastructure, and inequities in healthcare delivery exacerbate these issues, particularly in rural and resource-limited areas [1,2]. The use of health information technology (HIT), particularly CDSSs, offers a pathway to address these challenges by enabling evidence-based, standardized decision making. India is known for its extensive ethnic and socioeconomic diversity, which presents both opportunities and challenges for delivering equitable healthcare. India’s cultural, religious, linguistic, and socioeconomic diversity present unique challenges in delivering equitable healthcare. The Indian healthcare system has long been challenged by variable quality of services, fragmented data, and restricted access to affordable care. The emergence of HITs offers renewed hope to clinicians regarding these critical challenges. In under-resourced regions, HIT offers the unique potential to bridge gaps in clinical capacity and decision making. Studies have shown that HIT often yields greater relative benefits in underdeveloped or resource-limited settings, where traditional healthcare infrastructure is weak. For instance, Meunier et al. (2023) noted that digital health interventions, including a CDSS, led to a 30% improvement in clinical guideline adherence in sub-Saharan Africa, compared to 15% in high-income countries [3]. This highlights the disproportionate potential of HIT, particularly tools like CDSSs and EHRs, to transform healthcare delivery and bridge access and quality gaps in low- and middle-income countries (LMICs) [1,4].

Over the last 20 years, health information systems and related technologies have been integrated into the delivery of healthcare services [5]. A CDSS is a form of HIT designed to assist healthcare providers in making or validating clinical decisions. When effectively designed and implemented, CDSSs can improve patient outcomes and the quality of care [6,7]. A CDSS supports health professionals in making informed decisions from diagnosis to treatment and in delivering both patient and preventive care.

CDSSs provide clinicians with person-specific information that is intelligently filtered and presented clearly and concisely, thereby reducing errors, enhancing efficiency, and improving the quality of healthcare [8]. CDSSs are a vital tool that supports evidence-based medicine and are utilized to augment healthcare processes through the centralization and standardization of data, diagnostic and therapeutic aids, consensus on best practices for ideal clinical conduct, setting standards, developing guidance systems, and knowledge transfer [3].

India’s healthcare sector is rapidly embracing IT-driven solutions, recognizing the potential to enhance service delivery and patient care [9]. Meanwhile, the Indian health informatics sector is expanding and diversifying, enabling CDSSs to support decision making, particularly in cases that require specialized medical expertise and those involving the assessment of multiple factors [10]. Nevertheless, CDSS implementations have encountered high failure rates with respect to end-user acceptance and sustained use [11].

Recent national digital initiatives in India demonstrate both the speed of progress and practical challenges of implementing CDSS-enabled care. The government announced 797.1 million Ayushman Bharat Health Accounts and 650.9 million connected health records under the Ayushman Bharat Digital Mission as of 28 July 2025 to establish systems for EHR integration and large-scale decision support [5]. The current review shows that CDSSs improve healthcare operations and disease management, with evidence for both EHR-integrated and stand-alone tools in different settings [6]. Sustained use by clinicians depends on human factors and sociotechnical fit; recent evidence indicates that trust in data-driven recommendations, professional identity dynamics, and workflow alignment determines long-term adoption in India’s diverse service environments [4,7].

The efficacy of an intervention cannot be evaluated unless it is used. Early failure is a critical challenge; therefore, healthcare organizations in India should prioritize identifying and addressing obstacles to implementation. Over the years, CDSSs have transformed into corporate AI-driven support systems. Mobile health applications can accommodate large datasets and advanced algorithms. Identifying priority use cases for CDSSs and incorporating stakeholder perspectives will facilitate success [8].

This review aimed to scrutinize the current utilization of CDSSs in Indian healthcare institutions and identify the primary implementation challenges associated with their adoption. The therapeutic benefits and operational advantages of CDSSs were analyzed, with proposals for addressing existing challenges and developing guidelines for the future expansion of CDSS usage. This review integrates existing data with expert perspectives to delineate the critical steps for the successful adoption of CDSSs in India’s healthcare system. This review aims to (1) examine the coverage and prevailing access to CDSSs in Indian healthcare settings, (2) identify key barriers to the adoption of CDSSs, (3) evaluate their potential benefits in improving healthcare delivery, and (4) propose strategies for the effective implementation of CDSSs based on national and international experiences, especially in LMICs.

## 2. Methodology

We conducted a narrative review to understand the utility of CDSSs in Indian healthcare settings and to identify potential barriers to their optimal implementation.

A search through medical databases such as PubMed, Scopus, Web of Science, and Google Scholar for relevant literature published through March 2025 was performed using the keywords ‘clinical decision support systems,’ ‘CDSS implementation’, ‘healthcare’, ‘electronic health records’, and ‘information technology.’ Only articles published in the English language were considered. We included studies involving patients receiving hospital-based care (population) where a CDSS was used (intervention), with or without a comparator (comparison), and those reporting clinical or operational outcomes (outcome) following the PICO criteria. The information was retrieved from clinical trials and observational and analytic studies, whereas case reports and case series were excluded. Additionally, we reviewed the official websites of Indian hospitals to identify announcements regarding CDSS deployments [12,13,14,15,16,17].

## 3. Benefits of CDSSs in Patient Care

A meta-analysis by Chen et al. (2022) found that implementing CDSSs across LMICs reduced diagnostic errors by 12–15% and improved patient outcomes by 10% [18]. In India, pilot studies at AIIMS and PGIMER reported cost savings of up to 18% in medication management through CDSS integration. White et al. (2023) estimated that CDSS implementation in hospitals can potentially yield a return on investment (ROI) of 1.5 to 2.8 times within the first three years, primarily through reduced adverse drug events and shorter hospital stays [19].

**Figure 1:**
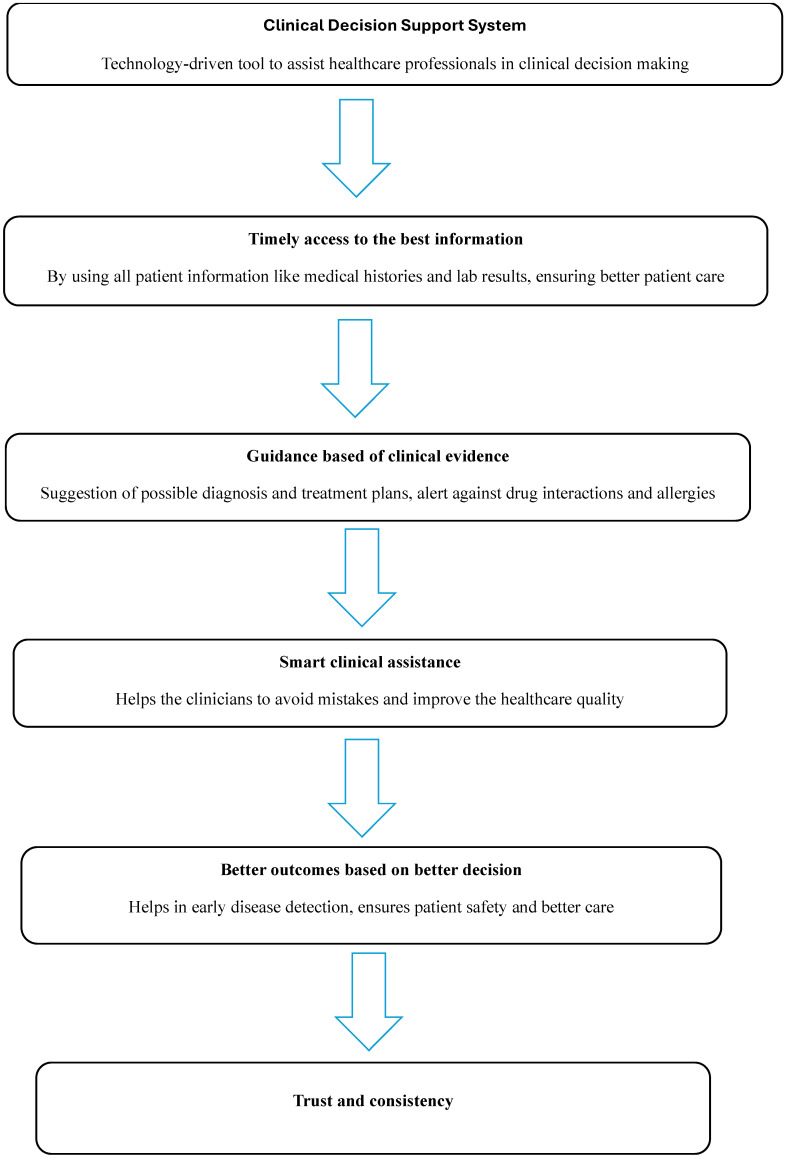
Flowchart showing the utility of CDSSs.

Complex clinical tasks pose challenges to decision making owing to uncertainties and associated risks. Decisions are grounded in evidence from multiple sources (e.g., research and clinical data) and informed by patients’ needs and preferences. CDSSs overcome the complexity and ambiguity in healthcare, enabling well-informed, timely decisions facilitated by digitally compiled, frequently updated clinical evidence. CDSSs also improve the clinical decision-making processes of healthcare providers. CDSSs provide diagnostic aids, drug interaction (DI) checks, alerts, and reminders, thereby improving cost-effectiveness, quality of care, and patient safety [18,19,20]. As drug-related problems (DRPs) are a major concern in patient care, CDSSs play a major role in mitigating DRPs by providing early alerts and notifications. CDSSs also contribute to public health by providing early alerts and supporting drug selection and personalized therapy [4]. Figure 1 depicts the utility of CDSSs.

A CDSS provides a platform for clinicians to efficiently manage patient data at the point of care [21], thereby reducing costs and improving patient care [22,23]. At the point of care, CDSSs support diagnosis and prescribing, reduce adverse drug reactions (ADRs), and assist with ordering tests and interpreting laboratory results, while minimizing costs [24,25,26]. The applications of CDSSs in clinical practice are vast, and most of their benefits are listed in Table 1.

## 4. Importance of CDSSs in Indian Healthcare

The growing population, rising life expectancy, and rural–urban divide in healthcare services have increased India’s urgent need to embrace CDSSs. By disseminating guidelines, cancer databases, and consultancy services to primary care doctors, nurse-led clinics, care homes, and emergency services nationwide, CDSSs can present risk stratification, therapeutic options, and prognosis to a potentially larger audience, thereby improving the allocative efficiency of evidence-based healthcare services at the point of care. Given the reach of the Internet today, CDSSs can minimize geographical disparities between rural and urban settings. India is among the largest software developers globally; a partnership between healthcare providers and software vendors stands to benefit from the development, deployment, and use of CDSSs, especially in managing non-communicable diseases that require a multidisciplinary team approach [27,28].

## 5. Adoption of CDSSs in Indian Healthcare Settings

A comprehensive CDSS-enabled healthcare IT system is unavailable in most Indian hospitals, possibly because of widespread misunderstandings about the limited scope for customization and limited institutional buy-in. Hospitals that utilize CDSSs are limited; a few examples are listed in Table 2.

An Integrated Tracking, Referral, Electronic Decision Support, and Care Coordination (I-TREC) model of care was established in government hospitals, focusing on non-communicable diseases under the comprehensive primary healthcare initiative. I-TREC has imparted evidence-based recommendations for treating hypertension and diabetes. Healthcare can feed anonymized patient data to the CDSS platform, which then processes and generates management plans [29]. The National Health Authority, India, with the All-India Institute of Medical Sciences and the Center for Chronic Disease Control, developed and presented CDSS tools for the Ayushman Bharat Digital Mission. Remarkably, the CDSS tool is free to use and can generate personalized clinical plans, promote drug and dose adjustments, identify patient risks, facilitate diagnosis, aid follow-up, and monitor potential contraindications [30]. Despite being offered free of charge, physicians were skeptical about the validity of CDSSs. Zhuliany Huang et al. found differences in confidence levels among physicians in India and Singapore. Fear of loss of autonomy, loss of clinical skills, and difficulty in use were among these concerns [31].

## 6. Challenges and Barriers to CDSS Implementation in India

A 2023 report by the National Health Authority, India, states that only 23% of Indian hospitals have interoperable EHR systems and less than 15% integrate CDSS tools effectively into clinical workflows [32]. Misro et al. (2023) also found that over 60% of healthcare providers cited lack of interoperability as a major barrier to CDSS adoption [33].

While CDSSs enhance healthcare quality, reduce medication errors, and promote evidence-based practices, India faces significant challenges and barriers to adopting these systems. These obstacles constitute four main areas.

### 6.1. Technological Challenges

Emerging technologies support CDSS development and demonstration, from electronic or patient registries to other electronic data sources, to guide the CDSS relevant to a clinician’s and patient’s context during clinical decision making. The absence of such technological infrastructure in resource-limited settings necessitates substantial investment and maintenance, which is a significant impediment to implementing CDSSs. Unfortunately, most healthcare institutions in India provide little or no infrastructure support for CDSSs. As with any other resource-limited region, strengthening data capture and compilation is essential for India to decrease uncertainty in evidence-based medicine and to inform policy formulation. Strengthening data capture, system integration, and digital infrastructure remains a foundational step for the success of CDSSs in India. Many institutions operate without basic health IT systems, which exacerbates implementation gaps and limits real-time clinical decision making.

The EHR program, funded partially by the Ministry of Health and Family Welfare, India, outlines requirements and proposes standards of EHRs to make them “personalised, portable, and valuable to the patient, doctor, hospital, and to society”. It is limited to the private sector and many government and teaching hospitals in the states, making it inaccessible to a vast majority of other healthcare facilities operating in India [34]. Rural health institutions find it more challenging to implement CDSSs than those in urban areas. Rural challenges encompass a range of concerns, including technological, organizational, physician-related, financial, and other issues [35,36].

Current hurdles include a lack of suitable infrastructure, interoperability issues, concerns regarding data quality and accessibility, and concerns regarding data privacy and cybersecurity. Inadequate digital infrastructure remains one of the most pressing barriers to the adoption of CDSSs in India. Many healthcare institutions still lack the foundational systems required to effectively capture, store, and integrate patient data. Without investment in interoperable EHR platforms and consistent data standards, real-time clinical decision making will remain out of reach [37]. Data quality and availability are other primary concerns. CDSS performance relies on correct and up-to-date patient data. However, healthcare organizations in India face difficulties in collecting complete and well-characterized data, which compromises the structural competence of the CDSS [38]. Other challenges include data protection and patient confidentiality. Transitions to digital systems increase the risk of data-security breaches and pose privacy threats [33].

### 6.2. Financial Challenges

Financial constraints, including high implementation and maintenance costs, training fees, and insufficient government funding, make implementing CDSSs in India challenging. A CDSS requires significant investment in hardware, software, and human resources. Most public healthcare institutions in India face financial constraints that limit their ability to acquire health technologies [19]. Regular CDSS maintenance and user training increase the budgetary burden [39]. However, the limited government funding is another concern. Unlike other countries, India does not rely primarily on government funding to embrace health IT. Few funding options exist for deploying CDSSs in India’s private and public facilities [23].

Indian healthcare is generally considered more cost effective than healthcare systems in the US and European countries. Therefore, CDSS tools developed in developed countries cannot be used as models in developing countries because of the lack of data and improper standardization. Cost-effective research may help to build a CDSS tool that can be implemented globally. Investment in such research is worthwhile and can improve healthcare delivery and quality. Economic evaluations of CDSS tools can encourage healthcare administrators and government agencies to utilize and implement the available tools, provided they are performed and published. Most developing countries do not conduct economic analyses related to the CDSS tools they implement. To be economical, advocates should develop network-based tools, web-based applications, and shared systems [19].

The costs of maintaining an organization’s infrastructure should be factored into the budget. The Indian healthcare system is neither organized nor follows standard practices. Without continuous effort towards improvement and follow-up, CDSSs could fail. Information technology may not reduce the number of healthcare professionals but can eliminate redundant services, reduce the likelihood of diagnostic errors, and shorten a patient’s hospital stay. Hence, when developing tools, their competitiveness in the market should be assessed to plan a more effective economic model for promoting CDSS tools [39].

### 6.3. Data Quality and Availability

This is one of the most important challenges in designing and implementing CDSSs in Indian healthcare settings. The majority of healthcare centers in India operate stand-alone or have limited interoperability among their various departments. This could also be because multiple private vendors provide IT-enabled solutions to these organizations, which do not support data exchange between these various systems. In such a scenario, the data available for analyses are inconsistent over the entire patient care process. This may be only a portion of the care process offered to a patient in this organization. This leads to various data quality issues, including missing, noisy, incomplete, and inconsistent data. The availability of accurate and comprehensive patient-specific data is crucial for the success of CDSSs in providing reliable and useful recommendations [38,40].

### 6.4. Regulatory and Legal Barriers

The adoption of CDSSs in India may encounter numerous regulatory and legal challenges. The lack of clarity in governmental policies has resulted in inconsistent CDSS adoption in Indian healthcare institutions [41]. Healthcare professionals have also raised concerns regarding the irresponsibility and liability of CDSSs. Physicians are concerned about the potential legal implications of poor patient outcomes resulting from the use of CDSS guidance [22]. Social responsibility is another concern. Clear policies must address ethical concerns regarding patient data ownership, informed consent, and AI systems [42].

### 6.5. Cultural, Professional, and Organizational Barriers

Clinicians may resist CDSSs because of the perceived loss of autonomy, concerns about accountability, and fear of de-skilling or role displacement. Management may worry about workflow disruptions and power dynamics. In India’s traditional physician-centered system, where overwork increases the risk of medication errors, multidisciplinary collaboration and up-to-date IT tools such as CDSSs can enhance patient safety. Targeted training and clear governance can help build confidence and clarify responsibilities, thereby reducing resistance [11,27,36,43].

### 6.6. Integration with Electronic Health Records

One of the most important determinants of CDSS effectiveness is seamless integration with EHRs. However, the lack of common standards and poor interoperability remain major barriers to its development and adoption. Users cannot be expected to work across disconnected systems; tighter integration is needed to ensure consistent and effective use [35,37].

## 7. Suggestions for Improving CDSS Adoption in India

Countries such as Brazil and South Africa have made notable progress in CDSS adoption despite infrastructural constraints similar to India. Brazil’s national e-SUS platform integrates a CDSS for primary care, supported by government funding and training programs [44]. In South Africa, real-time adherence tracking via smart pillboxes supported clinical decisions, yielding 79% treatment success in new/relapsed and HIV-positive TB cases and 62% in rifampicin-resistant TB [45]. These examples highlight the importance of centralized policy, local customization, and capacity-building strategies that can inform India’s roadmap.

Strategic interventions that focus on technological, financial, regulatory, and institutional reforms are necessary to overcome the challenges of CDSS adoption in India.

### 7.1. Strengthening Technological Infrastructure

Investments in broadband Internet access, cloud-based data storage, and interoperability features of EHR systems will enable the implementation of CDSSs [34]. The availability of national interpretative standards for healthcare data will improve the usability of CDSSs in multiple hospitals [19]. Improvements in data encryption will reduce physical access to data and storage areas. Adherence to accepted standard practices in IT security is mandatory to safeguard patient information [39].

### 7.2. Financial and Policy Interventions

The government must subsidize rural hospitals and the public sector by integrating CDSSs [23]. Private healthcare and ICT companies have increased the state’s role in utilizing CDSSs at reduced costs [41]. Cost–benefit analyses and the evaluation of CDSS implementation significantly impact the technology financing readiness of various stakeholders [19].

### 7.3. Regulatory and Legal Reforms

A unified CDSS policy framework specifying integration, validation, and usage parameters can enhance adoption [44]. Delineating a CDSS’s responsibility in clinical decision support and defining liability limits can help resolve legal issues [22]. Policies regarding informed patient consent, data confidentiality, and the ethics of AI-based decision making must be established for responsible deployment of CDSSs [42].

### 7.4. Capacity Building and Training

Education-based programs for the entire territory must instruct medical doctors, nurses, and pharmacists on the application of CDSSs [11]. Teaching digital health and CDSSs must be part of the curriculum for medicine, pharmacy, nursing, and related fields [27]. To keep up with the changes in CDSS technology, regular workshops, such as refresher courses or online options, must be provided.

### 7.5. Strategic and Organizational Change

Motivating healthcare workers, including doctors, in the creation and execution stages of a CDSS can help improve its acceptance and usefulness [44]. Greater emphasis must be placed on real-life cases in which patients benefit from a CDSS to foster trust and its adoption [26]. Changing user experience and interface design is another concern. CDSSs must be designed to be easy to use and fit well into the working environment [36].

## 8. Conclusions

The adoption of a CDSS in India remains limited by technological, financial, regulatory, and cultural barriers. Nevertheless, CDSSs can significantly improve the quality and efficiency of care. Priority actions include workforce training, interoperable EHR infrastructure, and public awareness implemented through coordinated efforts among the government, providers, and technology partners. Policymakers should establish a national framework for CDSS implementation across public and private facilities to enhance outcomes and reduce the costs of medication errors.

## Figures and Tables

**Table 1:** Various applications of CDSSs in clinical practice.

Sl. No.	Category	Descriptions
1	Emergency and Critical Care Support	Early warning for sepsis, stroke, and cardiac arrest
		Triage decision support
		Ventilator management and respiratory monitoring
2	Laboratory and Radiology Decision Support	Automated lab result interpretation (e.g., critical value alerts)
		AI-assisted imaging analysis
		Radiology order appropriateness checks
3	Infectious Disease Management	ASP (e.g., antimicrobial therapy recommendations)
		Infection control alerts (e.g., MDR organisms and sepsis detection)
		Outbreak surveillance and early warning systems
4	Preventive Care and Public Health	Immunization reminders (e.g., pediatric and geriatric vaccines)
		Cancer screening alerts (e.g., mammograms and colonoscopy reminders)
		Smoking cessation and lifestyle modification suggestions
5	Personalized and Precision Medicine	Pharmacogenomics-based drug selection
		Oncology decision support for target therapies
		AI-driven risk prediction models for individualized treatment
6	Surgical and Anesthesia Support	Preoperative risk assessment tools
		Anesthesia dose calculation and DI alerts
		Postoperative complication risk prediction
7	Optimization and Administrative Support	Task automation and scheduling
		Clinical documentation assistance (e.g., voice-to-text transcription and structured note generation)
		Predictive analytics for hospital resource allocation
8	Geriatric Care and Fall Risk Prediction	Polypharmacy risk management
		Fall risk assessment and prevention strategies
		Cognitive impairment screening (e.g., dementia risk prediction)
9	Pediatric and Neonatal Care	Growth and development monitoring
		NICU support
		Pediatric drug dose calculation and alerts
10	Medication Management	DI alerts
		Allergy and ADR warnings
		Dose adjustment recommendations
		Duplicate therapy alerts
		Medication reconciliation
		Automated dispensing support
11	Diagnostic Assistance	CDT for differential diagnosis
		AI-powered image recognition for radiology and pathology
		Symptom checker tools for early disease detection
		Lab test interpretation and recommendations
12	Chronic Disease Management	Diabetes management (e.g., HbA1c monitoring and insulin dose adjustment)
		HTN monitoring and control recommendations
		COPD and asthma management
13	Clinical Guidelines	Integration of clinical practice guidelines
		Personalized treatment recommendations based on patient data
		Best-practice alerts (e.g., sepsis protocols and stroke management)

AI: artificial intelligence; ASP: antimicrobial stewardship; MDR: multidrug resistant; DI: drug interactions; ADR: adverse drug reaction; CDT: clinical decision trees; NICU: neonatal intensive care unit; HTN: hypertension; COPD: chronic obstructive pulmonary disorder.

**Table 2:** Healthcare settings utilizing CDSSs across India.

Sl. No.	Place	Health System
1	Bengaluru, Karnataka	Narayana Health
		Manipal Hospitals
		Cloudnine Hospitals
		Aster DM Healthcare
		HCG
		Sakra World Hospital
2	Chennai, Tamil Nadu	Apollo
3	Cochin, Kerala	AIMS
4	Telangana	KIMS
5	Mumbai	Tata Memorial Hospital
6	Gurgaon, Haryana	Medanta
7	Chandigarh (UT)	PGIMER
8	New Delhi	AIIMS
		Armed Forces
		Max Healthcare

HCG: Healthcare Global Ltd.; AIIMS: All India Institute of Medical Sciences; AIMS: Amrita Institute of Medical Sciences; KIMS: Krishna Institute of Medical Sciences; PGIMER: Postgraduate Institute of Medical Education and Research; UT: Union Territory.

## Data Availability

Data sharing is not applicable. No new data were created or analyzed in this study.

## Abbreviations

The following abbreviations are used in this manuscript:

ADRs	Adverse drug reactions
AI	Artificial intelligence
AIIMS	All India Institute of Medical Sciences
AIMS	Amrita Institute of Medical Sciences
ASP	Antimicrobial stewardship program
CDSS	Clinical Decision Support System
CDT	Clinical decision trees
COPD	Chronic obstructive pulmonary disorder
DI	Drug interactions
DRPs	Drug-related problems
HER	Electronic health records
HCG	HealthCare Global Ltd.
HIT	Health information technology
HTN	Hypertension
I-TREC	The Integrated Tracking, Referral, Electronic Decision Support, and Care Coordination
KIMS	Krishna institute of Medical Sciences
LMICs	Low-and middle-income countries
MDR	Multidrug resistant
NICU	Neonatal intensive care unit
PGIMER	Postgraduate Institute of Medical Education and Research
PICO	Patient, Intervention, Comparison, and Outcome
ROI	Return on investment
UT	Union Territory
